# Compulsive sexual behavior among male military veterans: Prevalence and associated clinical factors

**DOI:** 10.1556/JBA.3.2014.4.2

**Published:** 2014-12-18

**Authors:** PHILIP H. SMITH, MARC N. POTENZA, CAROLYN M. MAZURE, SHERRY A. MCKEE, CRYSTAL L. PARK, RANI A. HOFF

**Affiliations:** ^1^Epidemiology and Public Health, Yale University; ^2^Department of Psychiatry, Yale University School of Medicine; ^3^Department of Neurobiology, Yale University School of Medicine; ^4^Child Study Center, Yale University School of Medicine; ^5^Department of Psychology, University of Connecticut; ^6^Department of Veterans Affairs, VISN 1 Mental Illness Research Education and Clinical Care Center (MIRECC)

**Keywords:** compulsive sexual behavior, military, PTSD, child abuse, veterans, trauma

## Abstract

*Background and aims:* Compulsive sexual behavior (CSB) is highly prevalent among men, often co-occurring with psychiatric disorders and traumatic experiences. Psychiatric disorders and trauma are highly prevalent among military veterans, yet there is a paucity of research on CSB among military samples. The aim of this study was to examine the prevalence of and factors associated with CSB among male military veterans. *Methods:* Surveys were administered to veterans of Operations Iraqi Freedom, Enduring Freedom, or New Dawn at baseline (*n* = 258), 3 months (*n* = 194), and 6 months (*n* = 136). Bivariate analyses and Generalized Estimating Equations were utilized to estimate associations between CSB and the following variables: psychiatric co-morbidity, childhood physical or sexual trauma, pre- and post-deployment experiences, TV/ Internet usage, and sociodemographics. Associations between CSB and specific PTSD symptom clusters were also examined. *Results:* CSB was reported by 16.7% of the sample at baseline. Several variables were associated with CSB in bivariate analyses; however, only PTSD severity, childhood sexual trauma, and age remained significant in multivariable GEE models. The PTSD symptom cluster re-experiencing was most strongly associated with CSB. *Discussion:* This exploratory study suggests that CSB is prevalent amongst veterans returning from combat and is associated with childhood trauma and PTSD, particularly re-experiencing. *Conclusions:* Further study is needed to identify the mechanisms linking PTSD and CSB, define the context and severity of CSB in veterans, and examine the best ways to assess and treat CSB in VA clinical settings.

## INTRODUCTION

Compulsive sexual behavior (CSB) has been proposed to have two primary components: 1) a pattern of abnormally frequent paraphilic (e.g., fetishism, sadism, pedophilia) or normaphilic (e.g., sexual fantasies, sexual urges, intercourse, masturbation) thoughts and/or actions, and 2) significant distress and/or life problems associated with these thoughts/behaviors. Although CSB is not specifically recognized as a diagnosable clinical disorder by the DSM, Kafka (2010) proposed criteria for a hypersexual disorder that included the major components noted above, as well as additional elements such as perceived lack of control over these behaviors and engagement in hypersexual thoughts or behaviors in response to negative affect (Kafka, 2010). CSB as defined by these criteria has an estimated prevalence of 3–6% in the general U.S. population (Carnes, 1991; Coleman, 1992), and is associated with substantial emotional distress and social impairment. It has also been linked to potentially serious negative health and social outcomes such as sexually transmitted infections (Kalichman & Cain, 2004; Miner & Coleman, 2013) and sexual offenses (Kafka, 2003; Kingston & Bradford, 2013). Clinical data suggest that men are more likely to be treated for CSB (Bancroft & Vukadinovic, 2004; Kuzma & Black, 2008; Raymond, Coleman & Miner, 2003) and may have greater severity of CSB than women (Dodge, Reece, Cole & Sandfort, 2004; Kuzma & Black, 2008).

Mood, anxiety, substance-use, and personality disorders frequently co-occur with CSB (Black, Kehrberg, Flumerfelt & Schlosser, 1997; Carpenter, Reid, Garos & Najavits, 2013; Kafka & Prentky, 1994; Raymond, Grant, Kim & Coleman, 2002; Reid, 2007). Prevalence estimates for mood disorders among those with CSB have ranged from 39% to 81%, with those for anxiety disorders ranging from 46% to 96%, and those for substance-use disorders ranging from 46% to 71% (Kuzma & Black, 2008). Childhood sexual trauma (CST) may also predispose to CSB (prevalence estimates for childhood sexual abuse among those with CSB ranging from 30% to almost 80% [Black et al., 1997; Carries & Delmonico, 1996; Kafka & Prentky, 1992]), suggesting that traumatic experiences, early sexual encounters, or both may contribute to CSB. Impaired impulse control may contribute to CSB (Kor, Fogel, Reid & Potenza, 2013).

### Hypersexual behavior in military samples

An important research gap exists with respect to the paucity of CSB research amongst U.S. veterans and those deployed for active duty. CSB has been linked to psychiatric co-morbidity and deployed military personnel frequently experience psychiatric disorders (Schmied, Highfill-McRoy, Crain & Larson, 2013). Of particular concern for military populations is the relationship between compulsive sexual behavior and PTSD, a condition that has a substantially greater prevalence among military veterans compared to the general population. Researchers have noted some veterans may use sexual behaviors to cope with trauma (Howard, 2007), although the association between PTSD and CSB has not been extensively studied in military samples. CSB has also been linked to traumatic brain injury (TBI) (Bezeau, Bogod & Mateer, 2004), for which combat-exposed military personnel are at increased risk (Terrio et al., 2009). Therefore, due to the greater prevalence of these potential risk factors, one might expect to find greater prevalence of CSB among military veterans compared to non-military populations. CSB has been associated with spousal distress and family dysfunction (Reid, Carpenter & Draper, 2010; Reid, Carpenter, Draper & Manning, 2010), an issue that is also highly relevant in military samples, given the stresses of being separated from family during deployment and re-integrating with family upon return from deployment (Drummet, Coleman & Cable, 2003; Pincus, House, Christenson & Adler, 2001).

Despite these indicators that CSB may be important to study in military populations, there has yet to be a published study on the topic. This study presents an exploratory analysis of CSB in a sample of veteran men who were surveyed after returning from military deployment. The primary aim of this study was to identify both the prevalence of and potential risk factors for CSB among these men. We hypothesized that CSB would be associated with stress-related features and conditions (e.g., childhood trauma and PTSD) and with poorer adjustment during deployment and after returning from deployment.

## METHODS

### Procedure

Data were analyzed from the Survey of the Experiences of Returning Veterans (SERV) study, which was designed to examine the post-deployment experiences of veterans returning from Operation Iraqi Freedom (OIF), Operation Enduring Freedom (OEF) or Operation New Dawn (OND). Participants were eligible for the study if they had served in Iraq, Afghanistan, or surrounding areas or waters. Participants were recruited through multiple outlets, including the Internet (e.g., Facebook, YouTube), media outlets, Veterans Affairs (VA), resources such as listservs and closed circuit televisions in VA facilities, and word of mouth. Mean time since discharge at baseline for the sample was 3.32 years (*SD* = 2.45, range = 0.08–10.35). These veterans were then followed up at 3 and 6 months. Women were over-sampled in order to conduct gender comparisons; however, only men were included in the current investigation due to a small sample size for women reporting CSB (*n* = 7). For all waves of data collection, respondents were interviewed by trained staff using structured telephone interviews. These interviews lasted 60–80 minutes at baseline and 30–40 minutes at follow-up. Prior to the interview, participants were mailed a booklet with response scales in order to allow them to follow along with the interview more easily.

### Participants

The sample of men in this analysis was 258. Of these, 75.2% completed the 3-month interview and 52.7% completed the 6-month interview. Some of those who had not completed the follow-up interviews had yet to reach the time-window for the interview, as data collection for the study is on-going. The use of all three waves of data, although incomplete, allowed us to take advantage of all available information and examine change over time using Generalized Estimating Equations (see *Statistical analyses* section, below).

At the time of data analyses, 14.0% were lost to follow-up at 3 months and 15.2% were lost to follow-up at 6 months. At 3 months, 22.2% of those lost to attrition reported CSB at baseline, compared to 15.9% of those retained. At 6 months, 21.1% of those lost to attrition reported CSB at baseline, compared to 16.1% of those retained. Neither of these differences were statistically significant (*p* > 0.05). A greater percentage of those lost to follow-up at both 3 and 6 months were of non-White/Caucasian race/ethnicity (*p* < 0.05). There were no other differences based on sociodemographic characteristics.

### Measures

The primary aims of this study were to assess the prevalence of CSB among a sample of returning male veterans and to assess sociodemographic and clinical correlates of CSB, including potential risk and protective factors. Post-hoc analyses examined whether the sub-sample of veterans meeting diagnostic criteria for PTSD differed in the prevalence of CSB by different PTSD symptom clusters. Variables are grouped and described below.

### Compulsive sexual behavior

CSB was the primary dependent variable of interest. CSB was measured using items from the Minnesota Impulsive Disorder Inventory (MIDI; Grant, 2008) at baseline, 3 months, and 6 months. The CSB portion of this scale consists of 2 items, answered yes or no. Respondents were considered to have CSB if they responded affirmatively to either of the following two items: “Do you or others that you know think that you have a problem with being overly preoccupied with some aspect of your sexuality or being overly sexually active?” or “Do you have frequent sexual fantasies, urges, or repetitive behaviors which you feel are out of your control or cause you distress?” The MIDI has demonstrated very good validity in comparison to clinician diagnoses, in both adolescent and adult psychiatric populations (Grant, Levine, Kim & Potenza, 2005; Grant, Williams & Potenza, 2007).

### Sociodemographic characteristics

Age, education, income, marital status, and race/ethnicity were assessed at baseline. Education was coded to a binary variable, representing either less than or equal to or greater than high-school (75.1% ≥ high-school). Income was examined as a continuous ordinal variable, with 6 categories ranging from under $20,000 to $100,000 or more. Mean income for this sample was 3.12 (approximately $35,000 to $50,000), with a standard deviation of 1.58 and a range of 1 to 6 (<$20,000 to ≥ $100,000). Marital status was coded into three groups: 1) married or living with someone (50.8%), 2) divorced/widowed/separated (31.0%), and 3) never married (18.2%). Race/ethnicity was grouped into White/Caucasian, non-Hispanic (67.2%), Black/African-American, non-Hispanic (9.0%), Other, non-Hispanic (7.4%), and Hispanic (16.4%). Respondents could endorse more than one race/ethnicity category.

### Sociodemographic characteristics

Psychiatric comorbidity was measured using specific scales, each measured at baseline, 3 months, and 6 months. Depression was measured with the Primary Care Evaluation of Mental Disorders (PRIME-MD) (Spitzer, Kroenke & Williams, 1999). Anxiety disorders (generalized anxiety, panic disorder, and agoraphobia) were measured with the anxiety module of the Alcohol Use Disorder and Associated Disabilities Interview Schedule-IV (AUDADIS-IV; Grant & Dawson, 2000; Grant et al., 2003). Substance-use disorders (alcohol and drug abuse and dependence) were also measured using the AUDADIS-IV. PTSD was measured using the PTSD symptom checklist (PCL-civilian) (Wilkins, Lang & Norman, 2011). Symptom clusters from the PCL were calculated for further exploratory analysis. These clusters included symptoms of re-experiencing, emotional numbing, avoidance, and hyper-arousal (Greenberg et al., 2012; Simms, Watson & Doebbelling, 2002). Traumatic brain injury (TBI) was measured using the TBI screen employed by the VA to assess possible symptoms for follow-up. Items include a history of head injury, losing consciousness for 20 minutes or more, memory loss of the incident, and concussive symptoms such as headaches and dizziness.

### Childhood Trauma

Childhood trauma was categorized into two variables: 1) physical or emotional trauma, and 2) sexual trauma. Physical or emotional trauma was assessed with five items at baseline, each ranked from 1 to 5 (“never true” to “very often true”). Examples of items included “People in my family hit me so hard that it left me with bruises or marks,” and “People in my family said hurtful or insulting things to me.” Responses to these five items were averaged to create a single scale score (alpha = 0.87). This variable then was log-transformed to improve normality, and standardized to improve interpretability. Childhood sexual trauma was measured using a single item at baseline taken from the Deployment Risk and Resilience Inventory (DRRI; King, King, Vogt, Knight & Samper, 2006). Childhood sexual trauma (CST) was defined as an affirmative response to the following item: “[Prior to age 18] I experienced unwanted sexual activity as a result of force, threat of harm, or manipulation.”

### Military experiences

Military experiences during deployment (D) and post-deployment (PD) were also measured using the DRRI. For this analysis, the following scales were included: military preparation (D), deployment environment (D), unit support (D), unit relationships (D), combat experiences (D), social support after deployment (PD), and post-deployment stressors (PD). The scales that applied during deployment were measured at baseline only, and the scales for post-deployment experiences were assessed at baseline, 3 months, and 6 months.

### Television and Internet usage

Respondents were asked how many hours per day they watched television, and how many hours per week they used the Internet, at baseline, 3 months, and 6 months. Responses were categorized based on tertiles for both items. For television use, this resulted in the following categories: 0–1, 1–2, and >2 hours per day. For Internet use, the categories were: <7, 7–14, and >14 hours per week.

### Statistical analysis

All analyses were conducted using Stata Statistical Software version 13.0. First, we descriptively examined differences between those with and without CSB. We determined the significance of differences for all sociodemographic variables, potential risk factors, and potential protective factors using *t*-tests and chi-square tests of independence. Given the large number of variables examined in this study, only those for which we found differences at the bivariate level with a *p*-value <0.10 were included in multivariate analyses. We used a less-stringent cut-off of 0.10 due to the exploratory nature of the investigation, as well as the potential for significant associations to be masked by third-variable effects. Multivariable modeling was conducted using generalized estimating equations (GEE). This modeling approach appropriately handles both the non-independence among observations resulting from the longitudinal nature of the data and missing data resulting from attrition and non-response. GEE modeling requires data to be missing completely at random (MCAR). In other words, GEE requires that missingness does not depend on covariates or outcomes. As previously noted, missingness at 3 months and 6 months was unrelated to all study variables of interest, with the exception of small differences by race/ethnicity.

Models were specified using a binomial distribution for our CSB outcome variable and a logit link. We used full-maximum likelihood estimation, specifying an auto-regressive (1st order) working correlation matrix and estimated robust standard errors. We first examined a model with time as the only independent variable. We then added in other covariates and included those factors that were significant at *p* < 0.05 in a final model. Covariates measured at baseline were included in the models as time-invariant and those measured at all three time-points were included as time-varying. These models produced adjusted, time-averaged cross-sectional associations between CSB and other variables of interest, accounting for autocorrelation between time-points. Effect sizes are presented as odds ratios for categories in relation to a reference group or for every one point increase in the independent variable.

Pos-hoc analyses of the sub-sample of men with PTSD examined associations between specific PTSD symptom clusters and CSB. Indicator variables for each symptom cluster were added as independent variables in a longitudinal GEE model predicting CSB.

### Ethics

The procedures were carried out in accordance with the Declaration of Helsinki. The Institutional Review Board of the Department of Veterans Affairs approved the study. All subjects were informed about the study and all provided written informed consent.

## RESULTS

The prevalence of CSB in this sample at baseline was 16.7%. The prevalence dropped slightly at the 3-month interview (15.5%), and then dropped to 8.8% at 6-months follow-up. The difference in prevalence between baseline and 6 months was statistically significant (*p* < 0.05). This difference was not due to differential attrition – the difference between baseline and 6-month follow-up remained significant among those who had thus far completed all three waves of data collection. Further, rates of loss to follow-up did not differ between those with and without CSB at wave 1 (46.3% vs. 51.2% for those with and without CSB at wave 1, respectively; *p* = 0.56). Regarding consistency of CSB over time, 58% of those with CSB at wave 1 also identified with CSB at wave 2, and 36% also identified with CSB at wave 3. Of those with CSB at wave 2, 36% also identified with CSB at wave 3. Approximately 4% of the wave 3 sample was identified with CSB at all 3 time points.

Differences between men with and without CSB, at baseline, are displayed in Table 1. Men with CSB were more likely to be older (CSB, mean = 37.2yrs, *SD* = 15.0; no CSB, mean = 33.3yrs, *SD* = 8.2; *p* < 0.05), and of minority race/ethnicity (% White/Caucasian: CSB = 50.0; no CSB = 70.4; *p* < 0.05). Bivariate associations between CSB and both CST and childhood physical/emotional trauma were non-significant (*p* > 0.05). CST met the *p*-value threshold for further multivariable analyses (*p* = 0.075). All but one respondent of those with CSB were diagnosed with PTSD (97.7%), and those with CSB had significantly higher PCL scores (i.e., PTSD severity; *p* < 0.05). Men with CSB scored higher on the DRRI measure of relationship quality during deployment (*p* < 0.05), as well as on the DRRI post-deployment stressors scale (*p* < 0.05). Both findings are indicative of more negative experiences (i.e., poor relationship quality and more stressors).

**Table 1.** Baseline descriptive comparison of those with and without compulsive sexual behavior

	Compulsive sexual behavior			
	No (*n* = 184) *n* (%) or Mean * (SD)*	Yes (*n* = 36) *n* (%) or Mean * (SD)*	Effect size (Cohen’s *d* or OR)^b^	*p*-value
*Sociodemographics*				
Age	33.3 (8.2)	37.2 (15.0)	–0.30	**0.016**
Race/ethnicity				
White/Caucasian, non-Hispanic	150 (70.4)	21 (50.0)	Ref.	
Black/African-American, non-Hispanic	14 (7.0)	8 (19.1)	4.08	**0.005**
Other, non-Hispanic	15 (7.0)	4 (9.5)	1.90	0.290
Hispanic	33 (15.5)	9 (21.4)	1.95	0.132
Education level				
≤high-school	52 (24.4)	12 (27.9)	Ref.	
>high-school	160 (75.6)	31 (72.1)	0.84	0.641
Income (1–6)	3.1 (1.6)	3.3 (1.6)	–0.13	0.321
Marital status				
Married	104 (49.1)	25 (58.1)	Ref.	
Divorced/Separated/Widowed	67 (31.3)	13 (30.2)	0.81	0.569
Never married	42 (19.6)	5 (11.6)	0.50	0.179
Childhood trauma				
Childhood sexual trauma				
No	193 (91.6)	35 (81.4)	Ref.	
Yes	20 (9.4)	8 (18.6)	2.21	0.075
Childhood physical/emotional trauma				
(standardized)	–0.06 (0.90)	0.16 (0.95)	–0.18	0.150
*Psychiatric co-morbidity, non-substance use*				
PTSD (past mo.)				
No			–	–
Yes	27 (19.1)	1 (2.3)		
PCL score (range: 17–85)	185 (86.9)	42 (97.7)		
Depression (past mo.)	47.1 (18.7)	53.4 (14.5)	–0.26	**0.038**
No				
Yes	146 (68.7)	26 (60.5)	Ref.	
Anxiety (past mo.)	67 (31.3)	17 (39.5)	1.42	0.294
No				
Yes	126 (59.3)	19 (44.2)	Ref.	
Panic disorder (past yr.)	87 (40.7)	24 (55.8)	1.83	0.067
No				
Yes	140 (66.2)	27 (62.8)	Ref.	
	72 (33.8)	16 (37.2)	1.15	0.668
*Psychiatric co-morbidity, substance use*				
Alcohol dependence (past yr.)				
No	168 (79.0)	30 (69.8)	Ref.	
Yes	45 (21.0)	13 (30.2)	1.62	0.188
Drug dependence (past yr.)^a^				
No	202 (94.9)	41 (95.3)	Ref.	
Yes	11 (5.1)	2 (4.7)	0.90	0.894
*Other psychiatric variables*				
Probable TBI				
No	141 (67.0)	27 (62.8)	Ref.	
Yes	70 (33.0)	16 (37.2)	1.19	0.596
*TV/Internet use*				
Daily TV (hours)				
0–1	62 (29.0)	8 (18.6)	Ref.	
1.5–2	74 (35.1)	18 (41.9)	1.89	0.167
>2	77 (36.0)	17 (39.5)	1.71	0.244
Weekly Internet use (hours)				
<7	81 (38.5)	17 (39.5)	Ref.	
7–14	76 (36.7)	14 (32.6)	0.88	0.741
>14	55 (25.8)	12 (27.9)	1.04	0.926
*DRRI: Deployment*				
Deployment preparation (13–65)	44.8 (11.3)	42.0 (11.8)	0.24	0.145
Deployment environment (17–85)	49.7 (12.4)	52.5 (16.4)	–0.21	0.187
Unit support (20–100)	75.8 (17.9)	71.3 (15.3)	0.26	0.180
Unit relationships (16–80)	24.0 (6.4)	26.2 (8.8)	–0.25	**0.048**
Combat experiences (17–85)	24.1 (16.9)	23.1 (18.6)	0.06	0.729
Aftermath of combat (13–65)	22.5 (15.2)	22.0 (15.8)	0.04	0.840
*DRRI: Post-deployment*				
Social support (0–11)	3.8 (0.8)	3.7 (1.3)	0.07	0.674
Stressors (0–14)	3.8 (2.7)	4.8 (2.7)	–0.35	**0.034**

*Note:* Means *(SD)* and percentages are presented in columns. Statistically significant values in bold. Significance values are based on chi-square tests of independence or *t*-tests. Ranges for continuous variables are presented in parentheses following variable names.

^a^Drug dependence was not included in further GEE models due to insufficient sample size.

^b^For mean differences, Cohen’s *d*, CSB = yes vs. no; for categorical variables, odds ratios, odds of CSB among comparison group vs. odds of CSB among reference group.

Prior to adjusting for covariates, there was a significant association between time and CSB, whereby the proportion of respondents with CSB was significantly lower at 6 months post-deployment than at baseline (*p* < 0.05). This difference was no longer significant after the inclusion of other covariates in our model (*p* = 0.061). The final multivariable model is displayed in Table 2. Only three variables were statistically significant. Age was positively associated with CSB; every increase of 1 standard deviation in age was associated with a 30% increase in the odds of CSB (*p* < 0.05). Those with a history of CST had more than 3 times greater odds of CSB than did those without such trauma (OR = 3.17, 95% CI = 1.27–7.93). Finally, each standard deviation unit of increase in the PCL score (i.e., PTSD severity) was associated with a 55% increase in the odds of CSB (*p* < 0.05).

**Table 2.** Results from multivariable modeling: Factors associated with compulsive sexual behavior

	OR (95% CI)	*p*-value
Time		
Baseline	Ref.	
3 months	1.14 (0.3, 1.78)	0.560
6 months	0.53 (0.27, 1.03)	0.061
Age (standardized)	1.30 (1.07, 1.57)	**0.007**
Childhood sexual trauma	3.17 (1.27, 7.93)	**0.014**
Childhood physical trauma	2.38 (0.97, 5.82)	0.058
PTSD symptom severity (standardized)	1.55 (1.12, 2.12)	**0.006**

*Note:* Statistically significant values in bold. Based on GEE modeling, specifying binomial family, logit link, AR 1 correlation structure, and robust standard errors.

In our final model, we examined associations between specific PTSD symptom clusters and CSB among those with a PTSD diagnosis. The results of this model are displayed in Table 3. The re-experiencing symptom cluster was the only cluster significantly associated with CSB (*p* < 0.05). A 1-standard-deviation increase in the re-experiencing symptoms was associated with 87% greater odds of CSB.

**Table 3.** Associations between specific PTSD symptom clusters and compulsive sexual behavior among those with a PTSD diagnosis

	OR (95% CI)	*p*-value
Re-experiencing	1.87 (1.05, 3.31)	**0.032**
Avoidance	1.09 (0.71, 1.68)	0.684
Emotional numbing	0.96 (0.59, 1.53)	0.849
Hyper-arousal	0.71 (0.46, 1.12)	0.142

*Note:* Statistically significant values in bold. Based on GEE modeling, specifying binomial family, logit link, AR 1 correlation structure, and robust standard errors. All symptom cluster variables were standardized prior to analyses.

## DISCUSSION

This study is the first to examine CSB in a longitudinal sample of male veterans recently returning to civilian life after deployment. Several important conclusions can be drawn from these analyses. First, the prevalence of CSB, although it dropped over the course of follow-up, appeared considerably higher than published population estimates for CSB, suggesting that male veterans may be at particularly high risk for CSB. Secondly, increasing age and traumatic experiences, particularly childhood sexual and physical trauma as well as PTSD symptoms resulting from either combat or other trauma exposure, were significantly associated with CSB. Finally, among those with PTSD, re-experiencing symptoms specifically were associated with CSB. CSB may be an important clinical target in its own right, and improving PTSD symptoms may be beneficial for reducing CSB. It may also be important to address CSB in the context of PTSD symptoms, if CSB is used as an avoidance coping strategy (Howard, 2007).

### CSB prevalence and sociodemographic findings

CSB was more prevalent in this sample of male veterans than might be expected given published population prevalence estimates. At baseline, we found that 16.7% of the studied sample met criteria for CSB, a percentage several fold higher than the 3–6% rate reported in the general population (Carnes, 1991; Coleman, 1992). However, after 6 months, the percentage of CSB had declined to 8.8%, which is closer to the higher end of general population estimates. This effect was not likely due to attrition; the difference between baseline and 6-month follow-up remained significant when considering only those who completed all three waves of data collection, and loss to follow-up was approximately equal for those with and without CSB at wave 1. Rather, between waves 1 and 2, there were roughly equal numbers of remitted cases of CSB and newly identified cases of CSB, while between waves 2 and 3, the number of remitted cases exceeded the number of newly identified cases, resulting in a drop in prevalence. One possible explanation for this fluctuation in prevalence is that the two-item screening measure captured less severe cases of CSB, which are likely to be less stable over time. In the current study, only 3.7% of the wave 3 sample was identified with CSB at all three time-points. This pattern of chronicity (more stable for some, more episodic for others), however, is consistent with previous research on CSB, although few studies have examined the natural history of CSB (Kuzma & Black, 2008).

In bivariate and adjusted analyses, age was significantly associated with greater likelihood of CSB. Older individuals may find frequent sexual compulsions more distressing than younger individuals due to perceptions of normative behavior for specific age groups and family responsibilities. Minority race/ethnicity and lower education were also associated with greater likelihood of CSB in bivariate analyses, although these associations were no longer significant in the multivariable model. These findings suggest CSB may be a particularly concerning issue among individuals of minority status and with lower education, possibly due to greater frequency of traumatic experiences and more severe PTSD.

### Main hypotheses

We hypothesized that CSB would be associated with stress-related features and conditions (e.g., childhood trauma and PTSD) and with poorer adjustment during deployment and after returning from deployment. These hypotheses were generally confirmed. Although previous studies have found links between several forms of psychiatric co-morbidity and CSB, including mood disorders, anxiety disorders, and substance-use disorders (Black et al., 1997; Kafka & Prentky, 1994; Raymond et al., 2002; Reid, 2007), in the current study we found a significant association only for PTSD. Nearly all of those with CSB had a PTSD diagnosis at baseline (97.7%), and there was a significant and positive association between PTSD severity and CSB after adjusting for covariates. This finding is consistent with previous research. Blain, Muench, Morgenstern and Parsons (2012), examining potential risk factors for CSB in a sample of gay and bisexual men, also found that PTSD was associated with CSB, while depression and anxiety symptoms were not associated with CSB (Blain et al., 2012).

The association between PTSD and CSB may be particularly relevant for military samples, given the high prevalence of PTSD within military groups. In order to explore facets of PTSD linked with CSB, we examined associations with specific PTSD symptom clusters. We found CSB was significantly associated with the re-experiencing symptom cluster, but not other symptom clusters, among those with PTSD. This finding suggests that men may be engaging in CSB to help cope with the discomfort and stress related to re-experiencing trauma; this possibility warrants additional investigation. Further, our findings for specific symptom clusters appear different from the findings of Blain et al. (2012) that showed trend-level significant associations with a combined avoidance and emotional numbing symptom cluster. The explanation for this difference is not apparent, although it may be accounted for by variation in sample (men returning from military deployment versus a general sample of gay and bisexual men). The distribution of trauma types and CSB sub-types may vary between qualitatively different samples, potentially resulting in different mechanisms linking PTSD to CSB. Further research involving larger samples is needed to investigate potential mechanisms underlying the relationships between PTSD and CSB. From the current investigation, it does not appear that military veterans have unique risk factors for CSB; rather, they have high prevalence of possible risk factors for CSB that are also found in the general population.

We also found that those with a history of CST had more than 3 times greater odds of CSB than those without CST. This finding is consistent with previous studies showing high prevalence of CST among those with CSB (Black et al., 1997; Carries & Delmonico, 1996; Kafka & Prentky, 1992) and significant associations between CST and CSB (Blain et al., 2012). However, the percentage of those with CSB who reported a history of CST in this sample (18.6%) is substantially lower than figures found in other studies (approximately 30–80%), which may reflect differences in sample characteristics including trauma exposures. This interpretation is supported by the variability in findings from previous investigations, which suggests that variability in study samples may strongly influence the strengths of associations between CST and CSB. Researchers have proposed tension-reduction, or explanations related to dissociation during sex, to explain associations between CST and maladaptive adult sexual behavior (Hansen, Brown, Tsatkin, Zelgowski & Nightingale, 2012; Simpson, Griskevicius, Kuo, Sung & Collins, 2012). This may partly explain the association between CST and CSB found in this study; however, it is important to note that the association was significant above and beyond covariance with PTSD.

Unit relationships during deployment and post-deployment stressors were both associated with CSB in bivariate analyses. Multivariable analyses suggested these associations were accounted for by PTSD and CST, both of which are associated with social impairment and vulnerability to stress (Cloitre, Miranda, Stovall-McClough & Han, 2005; McLaughlin, Conron, Koenen & Gilman, 2010; Orsillo, Heimberg, Juster & Garrett, 1996). However, causal inference cannot be drawn from these findings. Overall, results suggest that in military samples, distal risk factors such as childhood trauma and psychiatric co-morbidity may be more relevant with regard to CSB than deployment/post-deployment experiences, including TBI.

A discussion of non-significant findings is also relevant. We did not find any associations between CSB and hours of TV watching or Internet use. This finding might suggest that the CSB might not be taking the form of extensive pornography viewing or online sexual behavior. However, because we do not have any information on the nature of the CSB activities (e.g. whether they were mostly fantasies, urges or actual sexual behavior), we cannot make this conclusion. Further examination of the context of CSB in military samples is needed.

### Implications

This investigation has important implications. Clinically, health care providers should be aware of CSB and assess for the condition. Awareness of CSB may facilitate referrals for assessment and treatment, which may reduce distress related to CSB, improve relationships and possibly prevent other problems such as sexual offending. Mental health clinicians, particularly those treating individuals with PTSD, should be aware of CSB as a potential co-morbidity with PTSD. Future studies are needed to examine how CSB may influence treatment for PTSD or vice-versa. It is possible that CSB may be improved by effective PTSD treatment; however, it is also possible that CSB may inhibit successful PTSD treatment by serving as an avoidance behavior. In PTSD treatment, patients are encouraged to reduce learned avoidance behaviors; if these behaviors include CSB, it may be necessary to pay special attention to sexual behavior specifically as an avoidance mechanism.

### Limitations

This study was subject to limitations inherent in conducting exploratory analyses. As noted, we did not collect information on the context of CSB; thus, it was not possible to examine mechanisms underlying the associations found in this study. Several possible predictor variables were examined, increasing the likelihood of finding statistical significance based on chance alone. However, the findings from the study are supported by evidence from previous research, bolstering their validity. Although our measure of CSB has been previously validated, the measure was limited to two items and did not include important dimensions of CSB such as the nature of the acts/thoughts and their frequency. As a result, these items may have resulted in overestimation of CSB prevalence. However, misclassification of some normative behavior as CSB would have likely biased associations towards the null. CSB was self-reported and prevalence may have been under-estimated as a result. The findings from this investigation are based on a convenience sample, and therefore may not generalize to other military samples. Our analyses examined associations, limiting the ability to make causal inference.

## CONCLUSIONS

In conclusion, this exploratory study suggests that CSB is prevalent amongst veterans returning from combat and is associated with childhood trauma and PTSD, particularly re-experiencing. Further study is needed to identify the mechanisms linking PTSD and CSB, define the context and severity of CSB in veterans, and examine the best ways to assess and treat CSB in VA clinical settings.
